# Circulating Nucleosomes as Potential Markers to Monitor COVID-19 Disease Progression

**DOI:** 10.3389/fmolb.2021.600881

**Published:** 2021-03-18

**Authors:** Etienne Cavalier, Julien Guiot, Katharina Lechner, Alexander Dutsch, Mark Eccleston, Marielle Herzog, Thomas Bygott, Adrian Schomburg, Theresa Kelly, Stefan Holdenrieder

**Affiliations:** ^1^ Clinical Chemistry Department, Centre Hospitalier Universitaire de Liege, Domaine Universitaire Du Sart-Tilman, Liège, Belgium; ^2^ Pneumology Department, Centre Hospitalier Universitaire de Liege, Domaine Universitaire Du Sart-Tilman, Liège, Belgium; ^3^ Department of Cardiology, German Heart Centre Munich, Technical University Munich, Munich, Germany; ^4^ DZHK (German Centre for Cardiovascular Research), Partner Site Munich, Munich Heart Alliance, Munich, Germany; ^5^ Belgian Volition SRL, Isnes, Belgium; ^6^ Eisbach Bio GmbH, Munich, Germany; ^7^ Department of Physiological Chemistry, Biomedical Center (BMC), Faculty of Medicine, LMU Munich, Munich, Germany; ^8^ Volition Germany GmbH, Gräfelfing, Germany; ^9^ Volition America, Austin, TX, United States; ^10^ Institute for Laboratory Medicine, German Heart Centre, Technical University Munich, Munich, Germany

**Keywords:** COVID-19, SARS nucleosomes, citrullination, neutrophil extracellular traps, NETosis, liquid biopsy, biomarkers

## Abstract

The severity of coronavirus disease 2019 (COVID-19) varies significantly with cases spanning from asymptomatic to lethal with a subset of individuals developing Severe Acute Respiratory Syndrome (SARS) and death from respiratory failure. To determine whether global nucleosome and citrullinated nucleosome levels were elevated in COVID-19 patients, we tested two independent cohorts of COVID-19 positive patients with quantitative nucleosome immunoassays and found that nucleosomes were highly elevated in plasma of COVID-19 patients with a severe course of the disease relative to healthy controls and that both histone 3.1 variant and citrullinated nucleosomes increase with disease severity. Elevated citrullination of circulating nucleosomes is indicative of neutrophil extracellular trap formation, neutrophil activation and NETosis in severely affected individuals. Importantly, using hospital setting (outpatient, inpatient or ICU) as a proxy for disease severity, nucleosome levels increased with disease severity and may serve as a guiding biomarker for treatment. Owing to the limited availability of mechanical ventilators and extracorporal membrane oxygenation (ECMO) equipment, there is an urgent need for effective tools to rapidly assess disease severity and guide treatment selection. Based on our studies of two independent cohorts of COVID-19 patients from Belgium and Germany, we suggest further investigation of circulating nucleosomes and citrullination as biomarkers for clinical triage, treatment allocation and clinical drug discovery.

## Introduction

The emergence and rapid progression of COVID-19 to pandemic status has placed substantial strain on international health care services. One characteristic of the disease is the development of life-threatening illness and alarming level of mortality (61.5%) in a subset of COVID-19 patients developing Severe Acute Respiratory Syndrome (SARS) resulting from infection with SARS-CoV-2 (Yan et al., 2020). Effective triage of these high-risk individuals represents an urgent and, so far, unmet clinical need to better direct appropriate resources for their care until herd immunity is established, protecting communities from future outbreaks.

Development of an effective triage strategy requires an understanding of the underlying pathophysiological processes defining the clinical course of COVID-19. Whilst reduction of viral replication is beneficial, it is not sufficient to avoid progression to severe and, in some case, critical illness and it is largely ineffective in later disease stages, which further underpins the necessity of predictive markers of severe disease course. Viral infection initiates cell death through various mechanisms (cell binding and entry, endosomal activation of TLR3 and gene expression) increasing the number of circulating nucleosomes in blood (Danthi, 2016). Thereby it has to be pointed out that the release of nucleosomes is a general mechanisms used by different cell types that are stressed or degraded by apoptosis, necrosis or other forms of cell death and high nucleosome levels are often associated with severe diseases and poor prognosis of the patients. However upon infection, neutrophils rapidly mobilize to sites of infection where they phagocytose pathogens and inactivate them through exposure to granular proteins (including lytic enzymes and antimicrobial proteins) and reactive oxygen species. In addition, viral infection can induce NETosis - a process where nucleosomal histone proteins H3, H2A, and H4 are hypercitrullinated by the nuclear enzyme PAD4. This promotes the massive decondensation of chromatin (Danthi, 2016; Mozzini and Girelli, 2020), resulting in the release of chromatin fragments into the extracellular matrix. These extended chromatin fibers are then released into the circulation and have been reported to immobilize viral particles via interaction with the DNA component of neutrophil extracellular traps (NET), thereby preventing dissemination and cellular uptake, as well as inactivating the virus by the action of the granular protein components including MPO and defensins (Saitoh et al., 2012). It was recently demonstrated that nucleosomes, derived from MNAse digestion of NET linker DNA, induce expression of the cytokine IL-1β cytokine expression from monocytes *in-vitro* and that citrullination enhances this proinflammatory signaling through enhanced binding to TLR4. Critically, DNAse degradation of the nucleosomal DNA neutralized their potency for monocyte activation and IL-1β induction, highlighting that citrullinated nucleosomes have higher proinflammatory activity relative to its subcomponents. DNA alone was insufficient to illicit a response. Above a critical threshold, nucleosome levels were toxic to monocytes and the authors concluded that this may provide a mechanism for immune suppression following NET aggregation (Tsourouktsoglou et al., 2020).

SARS-CoV-2, but also SARS-CoV and MERS-CoV (Barnes et al., 2020) were shown to initiate Neutrophil Extracellular Trap (NET) formation (Newton et al., 2016), and numerous studies report this as a key driver of pulmonary disease and associated fatality (Barnes et al., 2020; Monteil et al., 2020; Morath et al., 2020; Narasaraju et al., 2020; Zuo et al., 2020). The failure of the host’s immune system to clear the initial viral infection can lead to an over exuberant inflammatory response characterized by a cytokine storm, which is associated with excessive and sustained release of NETs. The resulting host-directed NETopathic events lead to Severe Acute Respiratory Syndrome (SARS) particularly in older males and those with underlying health conditions.

NET levels have been determined using free DNA quantification by fluorophore intercalation (Margraf et al., 2008), immunoassays based on their components including histone citrullination (Thålin et al., 2017), DNA/Myelo-peroxidase (MPO) combinations or DNA/Neutrophil elastase combinations (Kano et al., 2017), histone/DNA combinations (Sur Chowdhury et al., 2014) and histone/myeloperoxidase (Pieterse et al., 2018). Zuo et al. recently reported for the first time that cell free (cf)DNA and NET levels, measured as DNA-MPO, were elevated in COVID-19 patients receiving mechanical ventilation relative to patients breathing naturally (Zuo et al., 2020). However, citrullinated H3 or neutrophil counts were not elevated, prompting the authors to hypothesize that multiple NETosis pathways may be involved. Indeed, it has previously been reported that NETosis can occur by rapid (<30 min) “vital” pathways independent of reactive oxygen generation which may be without citrullination, or in a slightly slower (2 h) lytic (also known as suicidal), pathway mediated by oxidative triggering, via NADPH oxidase accompanied by citrullination (Castanheira and Kubes, 2019). Differentiation between these two pathways was highlighted as potentially important for disease specific NETopathic events (Kano et al., 2017; Holmes et al., 2019). Quantification of cfDNA in serum and plasma is a challenge, particularly with fluorophore intercalation methods like the one described by Zuo et al. (Zuo et al., 2020). The majority of cfDNA is stabilized within nucleosomes and the majority of linker DNA is rapidly degraded to leave mono and short poly nucleosomes via apoptotic and necrotic pathways (Holdenrieder et al., 2001). The potential proinflammatory activity of NET degradation products in the context of atherosclerosis was recently highlighted (Tsourouktsoglou et al., 2020). Furthermore, twisting of DNA around the histone core reduces intercalation efficiency and relative fluorescence intensity by a factor of six (Chen et al., 2007). Careful extraction of double stranded DNA is therefore required to achieve accurate quantification. On the other hand, immunoassays for the quantification of cell free nucleosomes have been described allowing simple measurements of cell free circulating nucleosomes and their post translational histone modifications (Holdenrieder et al., 2014; Bauden et al., 2015; Guiot et al., 2017; McAnena et al., 2017; Rahier et al., 2017). Nucleosomes have also been proposed as potential biomarkers for NET formation in plasma (Fuchs et al., 2010; Fuchs et al., 2012).

Thus far, no studies have been published on the association between cell free circulating nucleosome levels in patients with SARS-CoV-2 infection and their potential to stratify patients by disease severity. Based on the recent demonstration of the proinflammatory effects of nucleosomes and, in particular, citrullinated nucleosomes *in vitro*, we hypothesized that the levels of cell free circulating nucleosomes and their citrullinated derivatives may provide insight into disease severity in SARS-CoV-2 infected individuals. We present herein the first clinical evaluation of nucleosomes as potential biomarkers for disease severity in COVID-19.

## Results

**FIGURE 1:**
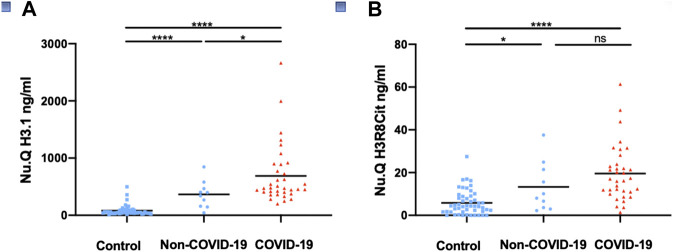
Detection of circulating nucleosomes associated with H3.1 or H3R8Cit in plasma of COVID-19 patients. Plasma from COVID-19 patients (*n* = 35), Non-COVID-19 patients (*n* = 10) and controls collected before the COVID -19 outbreak (*n* = 50) were assayed with Nu. Q H3.1 **(A)** or Nu.Q H3R8Cit **(B)**. COVID-19 patients were compared with Non-COVID-19 patients or control patients and Non-COVID-19 patients with controls by a Wilcoxon-Mann-Whitney test; ****p-value <0.001; *p-value <0.05, ns = no significant difference.

Circulating cell-free nucleosomes levels in COVID-19 patients. In a first cohort, we measured cell-free nucleosomes in samples taken from SARS-CoV2-positive patients (hereafter referred to as COVID-19; *n* = 35), SARS-CoV2-negative patients (hereafter referred to as Non-COVID-19; *n* = 10 as well as control patients (*n* = 50) collected before the COVID-19 outbreak at the University Hospital Center in Liege. Patients diagnosed with COVID-19 displayed significantly higher levels of cell free circulating nucleosomes containing histone variant H3.1 compared to healthy controls (mean COVID-19 = 689.2 ng/ml, control = 81.2 ng/ml; *p* < 0.0001; Figure 1A). H3R8 citrullinated nucleosomes were also significantly higher (mean COVID-19 = 20.6 ng/ml, control = 5.4 ng/ml; *p* < 0.0001; Figure 1B). Plasma H3.1 nucleosome levels were also significantly increased in hospitalized COVID-19 patients compared to hospitalized non-COVID-19 patients (Figure 1A; *p* < 0.05) whereas H3R8 citrullinated nucleosomes did not show any significant difference between the two groups (Figure 1B).

In samples taken within 5 days of admission, there was a trend toward higher H3.1 and H3R8Cit nucleosome levels in the 25 COVID-19 patients with more advanced disease suffering from severe acute respiratory syndrome (SARS) compared to the 10 COVID-19 patients without SARS, but the trend was not significant (Supplementary Figure S1). However, the two patients with the highest levels of H3.1 nucleosomes were COVID-19 with SARS and succumbed to the disease (Supplementary Figure S1A).

**FIGURE 2:**
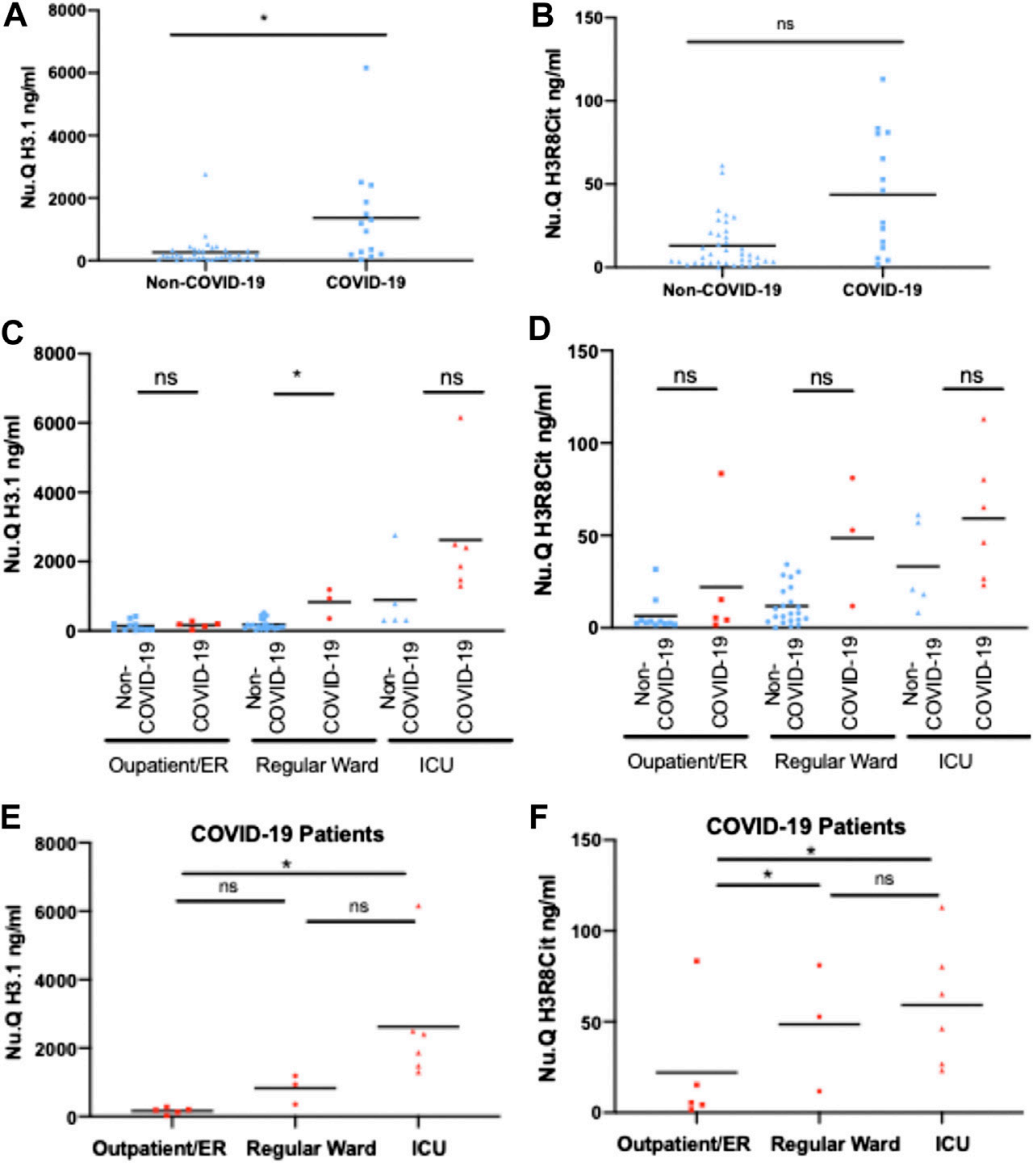
Circulating H3.1 Nucleosomes and H3R8 Citrullinated nucleosomes in plasma of COVID-19 and Non-COVID-19 patients admitted to ICU, Normal Ward or Outpatients/ER. Plasma concentrations of H3.1 Nucleosomes **(A)** and H3R8 Citrullinated nucleosomes **(B)** from 38 Non-COVID and 14 COVID-19 patients. H3.1 Nucleosomes **(C)** and H3R8 Citrullinated nucleosomes **(D)** levels from Non-COVID-19 and COVID-19 patient compared within each hospital setting (Outpatient/emergency room (ER), Regular Ward and Intensive Care Unit (ICU). Plasma from COVID-19 patients admitted to Intensive Care Unit (ICU) (*n* = 6); Regular ward (*n* = 3) or Outpatient/ER (*n* = 5) were assayed with H3.1 Nucleosome levels **(E)** and H3R8 Citrullinated nucleosomes **(F)**. Patients groups were compared by a Wilcoxon-Mann-Whitney test, *p-value <0.05, ns = no significant difference.

In a second, independent cohort collected at German Heart Center at the Technical University Hospital in Munich, we measured H3.1 and citrullinated nucleosome levels in samples taken from 14 COVID-19 patients and 38 Non-COVID-19 patients across different hospital settings: outpatient/emergency room (ER), regular ward or intensive care unit (ICU) as a proxy for disease severity: H3.1 nucleosome levels were significantly elevated in COVID-19 patients as compared with Non-Covid-19 patients (mean COVID-19 = 1,360.9 ng/ml, Non-COVID-19 = 263.4 ng/ml; *p* < 0.05; Figure 2A). For H3R8cit, a clear trend to higher values in COVID-19 patients was observed, too (mean COVID-19 = 43.7 ng/ml, Non-COVID-19 = 13.1 ng/ml; NS; Figure 2B). In addition, H3.1 and H3R8cit levels tended to be higher in COVID-19 patients within the different clinical settings, reaching the level of significance for H3.1 in the regular wards (Figures 2C,D). Within the group of COVID-19 patients, H3.1 and H3R8cit levels were significantly higher in all ICU patients relative to outpatient/ER patients (mean ICU = 2,623.1 ng/ml and 59.2 ng/ml, outpatient/ER = 166.4 ng/ml and 22 ng/ml; *p* < 0.05 for both; Figures 2E,F). H3R8cit values were also higher in patients at regular wards as compared with outpatient/ER patients (mean regular ward = 48.6 ng/ml, outpatient/ER = 22 ng/ml; *p* < 0.05 Figure 2F).

Thus, we can observe that H3.1 and H3R8cit nucleosome levels are higher in the COVID-19 patients with more severe disease. ICU patients and those on regular wards could be identified based on an H3.1 nucleosome threshold of [1,250 ng/mL] with the four non survivors in the Munich cohort having the highest overall H3.1 levels. Interestingly, H3.1 levels were also elevated in ICU Non-COVID-19 patients as compared with other hospital settings. Of note, during the pandemic the German Heart Center at the Technical University Hospital in Munich served as a referral clinic within southern Germany for severe COVID-19 cases and the majority of patients were referred from other hospital intensive care units to provide mechanical ventilation and Extracorporeal Membrane Oxygenation (ECMO).

**FIGURE 3:**
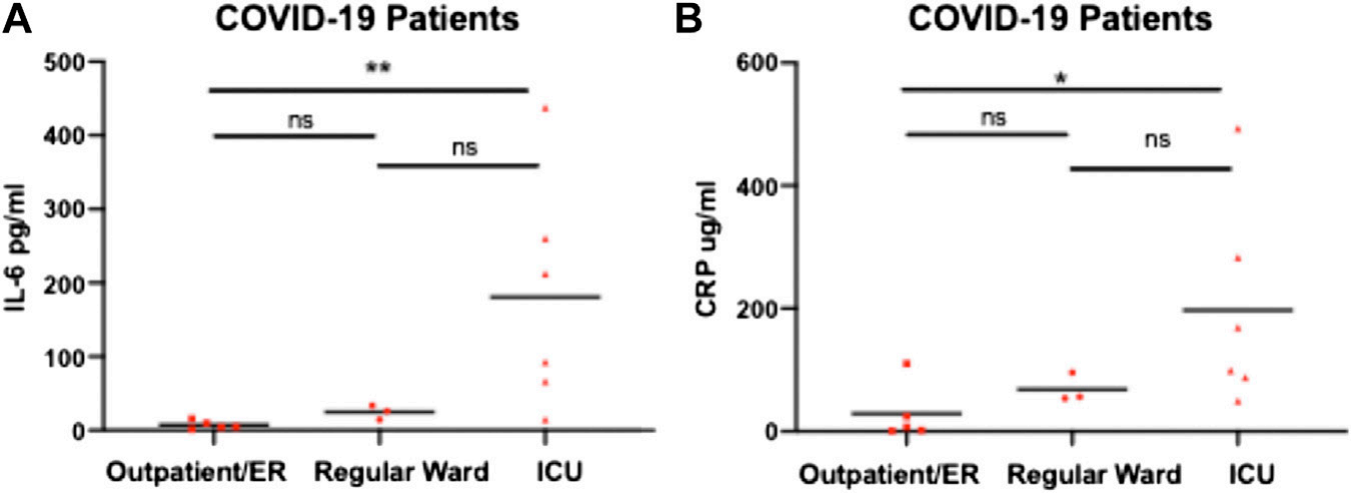
IL-6 and CRP levels in COVID-19 patients admitted to ICU, Normal Ward or Outpatient/ER. Plasma from COVID-19 patients admitted to Intensive Care Unit (ICU) (*n* = 6); Regular ward (*n* = 3) or Outpatient/emergency room (ER) (*n* = 5) were assayed with IL-6 **(A)** and CRP **(B)**. Patients groups were compared by a Wilcoxon-Mann-Whitney test, * *p*-value <0.05, ***p-value <0.01, ns = no significant difference.

To determine whether traditional biomarkers for inflammation showed the same elevated pattern with disease severity we measured IL-6 and CRP levels in COVID-19 patients across hospital settings (Figure 3). We found that for IL-6 and CRP showed a similar pattern as H3.1 nucleosomes in which there was a statistically significant difference in COVID-19 patients in ICU compared to the in outpatient/ER but not in the regular hospital ward. H3.1 and H3R8 citrullinated nucleosome levels in COVID-19 patients showed a significant correlation with each other in the Liege cohort (r = 0.588, *p* < 0.001), but not the Munich Cohort (r = 0.521, *p* = 0.056; Table 2). When correlating nucleosomal markers with more traditional inflammation associated biomarkers, we found that H3.1 nucleosome levels were correlated with IL-6 in both datasets (Liege: r = 0.504, *p* = 0.003; Munich: r = 0.855, *p* < 0.001) whereas H3.1 nucleosome levels correlated significantly with CRP in the Munich dataset only (r = 780, *p* < 0.001; Liege: r = 0.281, *p* = 0.102)). H3R8 citrullinated nucleosome levels did not correlate with either IL-6 or CRP in either dataset. The differences in the biomarker correlations could be reflective of the more advanced disease stage in the Munich cohort relative to the Liege cohort.

**TABLE 1:** Patient characteristics. Characteristics are given for COVID-19 and Non-COVID-19 patients from Liege and Munich.

Demographics	LIEGE	LIEGE	MUNICH	MUNICH
	COVID-19	Non-COVID-19	COVID-19	Non-COVID-19
Number	35^a^	10^a^	14	38
Female: Male	17:17		4:10	15:23
Age (years)	67.9 (range 29–89)		58.1 (range 26–79)	63.0 (range 31–90)
SARS positive: SARS negative	25:10			
Outpatient/ER: Regular ward: ICU			5:3:6	11:22:5
Comorbidities (number cases)				
High blood pressure	19		11	23
Diabetes	7		4	7
Chronic renal disease	8		3	6
Cardiopathy	8		4	13
COPD	1		0	3
Asthma	2		1	2
Respiratory status				
% Saturation oxygen	89.6 ± 6.7 (range 70–100)		92.6 ± 4.3 (range 85–97)^b^	95.7 ± 6.9 (range 60–100)^b^
Mechanical ventilation	2		6	0
Extracorporal MembraNe oxygenation			4	0

^a^ Demographic information one COVID positive patient in the Liege Cohort was not obtained; in addition to 10 Non-COVID-19 patients, 50 healthy controls collected prior to the COVID-19 pandemic were included in the analysis of the Liege cohort.

^b^ Oxygen status was not available for five and seven patients.

**TABLE 2:** Correlation between blood markers in COVID-19 patients. For the COVID-19 patients, correlations of between the different markers were assessed. Spearman’s rho correlation coefficients were calculated and are shown in the table. **correlation is significant at the 0.01 levels; *correlation is significant at the 0.05 levels.

	NU.Q H3.1		NU.Q H3R8CIT		IL6		CRP	
	Liege	Munich	Liege	Munich	Liege	Munich	Liege	Munich
Nu.Q H3.1	1	1	0.588**p < 0.001	0.521 *p* = 0.056	0.504**p = 0.003	0.855**p < 0.001	0.281 *p* = 0.102	0.780*p < 0.001
Nu.Q H3R8Cit			1	1	0.284 *p* = 0.109	0.319 *p* = 0.267	0.239 *p* = 0.166	0.169 *p* = 0.563
IL6					1	1	0.453**p < 0.001	0.851**p < 0.001
CRP							1	1

## Discussion

Our data demonstrate that cell free circulating nucleosome levels are significantly elevated in COVID-19 patients relative to controls examined prior to the COVID-19 outbreak. Nucleosome levels are further elevated in COVID-19 patients identified during non-SARS CoV 2 related hospital visits (as outpatients and in the emergency room setting) relative to those admitted to regular hospital wards for COVID-19. Nucleosome levels were highly elevated in COVID-19 patients requiring intensive care. This indicates a clear correlation between nucleosome levels and severity of COVID-19 and is consistent with previous reports of nucleosome levels predicting the disease severity based on cell free circulating nucleosomes as markers of cell death typically from apoptotic and necrotic pathways (Holdenrieder et al., 2001).

In the context of viral infection, multiple cell death pathways may be involved, including viral mediated lysis from infected cells. In addition, in patients who progress from mild to severe flu like symptoms to viral pneumonia and Acute Lung Injury (ALI) and SARS can experience multi organ failure which could be reflected in the circulating nucleosome levels. SARS in pathologic human coronavirus infection is associated with a catastrophic cytokine storm (Channappanavar and Perlman, 2017). The ability to predict disease severity, particularly in a resource limited medical scenario such as the SARS-CoV-19 pandemic, represents a clear and, so far, unmet clinical need.

Analysis of limited longitudinal cohorts from the early stages of the outbreak in China have focused on classical markers. Elevated lactate dehydrogenase (LDH) was shown to be associated with mortality and when combined with lymphocytes and high-sensitivity C-reactive protein (hs-CRP) was able to predict mortality with 90% accuracy up to 10 days in advance (Yan et al., 2020). In a second study, the chemokine RANTES (CCL5) was significantly elevated early in patients with mild disease compared to more advanced disease and the authors highlighted the potential prognostic power of combining CCL5 with IL-10 and IL-1RA which were associated with disease severity (Zhao et al., 2020). Interestingly IL-6 was only elevated in late stage severe cases, consistent with our data. Furthermore a variety of biomarkers, including levels of d-dimer, high-sensitivity cardiac troponin I, serum ferritin, lactate dehydrogenase, and IL-6 were significantly elevated in the most severe COVID-19 cases which resulted in death (Zhou et al., 2020).

The host-directed, immune response to an infection with SARS-CoV-2, which may be maladaptive in subgroups of patients with pre-existing medical conditions such as cardiometabolic disease, in large part determines clinical outcome. These bystander effects can be directly mediated by enzymatic activity associated with components of the NETs, including myeloperoxidase (Khan et al., 2018) and neutrophil elastase as noted in chronic kidney disease (Bronze-da-Rocha and Santos-Silva, 2018) or toxicity of degradation products including free histones (Marsman et al., 2016). Platelets may be involved in the process as they are associated with induction of NETs in Transfusion Related Acute Lung Injury (TRALI) which may result in hypoxia and bilateral pulmonary edema within 6 h of blood transfusion in a subgroup of patients (Caudrillier et al., 2012). These symptoms are also associated with COVID-19 severity.

The complex bidirectional interaction between neutrophils and cytokines make NETosis a likely mediator for severe symptoms that can be seen in response to SARS-CoV-2 infection, and in fact increased level of MPO-DNA that is associated with NETs was recently found in serum from COVID-19 patients (Zuo et al., 2020) and that plasma MPO-DNA complex levels increase with COVID-19 illness severity (Middleton et al., 2020). Once released, NETs can stimulate further cytokine production from macrophages which stimulates further NETosis generating a potential positive feedback loop and uncontrolled inflammation (Castanheira and Kubes, 2019). This is dramatically exemplified in multi organ failure that occurs in patients with sepsis (Margraf et al., 2008) and similar to what is seen in COVID-19. Therefore, neutrophils play a conflicted role in inflammation based on two juxtaposed aspects to neutrophil activation. The primary rapid innate immune response and inflammatory effects of neutrophil activation and NETosis are critical in the hosts ability to fight off pathogens but, sustained, can result in host directed bystander effects and cause substantial collateral damage to local tissue.

Citrullinated H3 protein assays have historically been used as markers for NETosis. However, a recent report of the interplay between proinflammatory signaling and toxicity of citrullinated nucleosomes as degradation products from neutrophil extracellular traps points to a potentially important role for “in context” detection of histone citrullination (Tsourouktsoglou et al., 2020). Intriguingly, while markers of NETosis were detected in COVID-19 patients and neutrophils from COVID-19 patients could trigger NETosis, the authors did not find a correlation between citrullinated H3 levels and COVID-19 (Zuo et al., 2020). We now demonstrate for the first time, that the level of citrullination in circulating nucleosomes is elevated and correlated with severity of COVID-19 in patients using a novel assay format specifically designed to measure Arginine R8 citrullination on histone three in the context of a nucleosome (see methods). It is possible that the effects of citrullination are specific to which residue is modified, however residue specific citrullination effects have not yet reported in the literature that we are aware of. Importantly our assay specifically measures citrullination in the context of nucleosomes and would not detect citrullination of H3 proteins in the absence of association with DNA and the other histones that make up an octamer. Thus, while cell death leads to increased nucleosome levels generally, the increase in citrullinated nucleosomes is consistent with a hyperinflammatory response associated with SARS-CoV-2 induced NETosis. This could also explain the differences we see in correlations across markers in the different cohorts. Consistent with clinical information, the Liege cohort represented a less advanced disease stage where circulating nucleosome are primarily derived from degraded NETs resulting in a correlation with citrullination. Meanwhile the Munich cohort represents a more advanced disease state where cell death also contributes non-citrullinated nucleosomes into circulation and elevated CRP levels (Mahajan et al., 2016).

Many underlying health conditions are reported to induce activation of neutrophils toward NETosis, which may explain why those with advanced age and pre-existing medical conditions/comorbidities are risk factors/predictors for COVID-19 related mortality. Elevated glucose levels resulted in NET release from neutrophils with type 1 and 2 diabetes (Wong et al., 2015) whilst increased capacity for NETosis in leukemia patients was correlated with priming by IL-8 (Podaza et al., 2017). Obesity is associated with chronic low-grade inflammation and obese mice show elevated NET levels in the lungs during influenza induced pneumonia (Moorthy et al., 2016). Diabetes and truncal obesity are both associated with increased risk of cardiovascular disease and NET levels have been shown to correlate with size of myocardial infarction and predict serious adverse cardiovascular events (Bonaventura et al., 2020). Intriguingly, diabetes, obesity and pre-existing cardiovascular disease are major risk factors for severe disease course in COVID-19 symptoms.

The presence of citrullinated, cell free circulating nucleosomes, as degradation products of NETs could potentiate the feedback loop and hyperimmune response triggering further NETosis via IL-1β signaling in individuals with activated neutrophils (Tsourouktsoglou et al., 2020). In addition, the toxicity of these citrullinated nucleosomes to monocytes, and potentially other cells, would contribute to further increases in general nucleosome levels in severe disease cases as apoptosis and necrosis occurs. This might offer some biological plausibility for the observation of increased mortality in patients with underlying health conditions. Circulating, citrullinated nucleosomes that are upstream of cytokine activation hold potential to serve as a potential prognostic marker to predict the severity of the disease course and could guide allocation of treatment resources and identify patients that could require more aggressive or targeted therapy.

### Future Perspective

The results described in this paper show the potential of using cell free circulating nucleosomes and epigenetically modified nucleosomes to monitor disease progression. Future studies will expand on the current study to examine circulating nucleosome levels, post translational modifications and correlations with additional clinical biomarkers and disease state in a longitudinal cohort where patients are identified early and sample collection is initiated prior to disease progression and throughout the course of treatment to correlate H3.1 nucleosome levels and citrullinated nucleosomes levels with disease progression. In addition, analytes which are traditionally used to measure NETs (MPO and NE) will also be run to further elucidate the role that NETosis plays in COVID-19, which could lead to new therapeutic targets.

## Methods

### Samples

In the Liege cohort plasma samples were evaluated from 50 healthy controls collected in 2019 prior to the COVID-19 outbreak together with 40 hospitalized COVID-19 confirmed patients (“Liege” cohort). 35 patients were tested positive in qRT PCR and five were diagnosed as COVID-19 positive by clinical observation and chest X ray and/or CT-scan, albeit being qRT-PCR negative. q-RT-PCR of oral swabs is used as the gold standard for diagnosis. Therefore, and for consistency between the two centers, q-RT-PCR negative patients were excluded in the statistical analyses. Among the 35 COVID-19 patients, 25 showed signs of Severe Acute Respiratory syndrome (SARS) defined as: oxygen saturation below 93 and/or shock or organ failure and/or hospitalization in an Intensive Care Unit (ICU); five were hospitalized in ICU and of those, two received mechanical ventilation for nine and ten days respectively. Ten qRT-PCR negative, Non-COVID-19 hospitalized patients were also included. For CHU of Liege, no specific approval was requested to the EC as a leaflet including the following statement is given to all admitted patients: according to the law of the December 19, 2008, any left-over of biological material collected from patients for their standard medical management and normally destroyed when all diagnostic analysis have been performed, can be used for validation of methods. The law authorizes such use except if the patient expressed an opposition when still alive (presumed consent). Written informed consent for participation was not required for this study in accordance with the national legislation and the institutional requirements.

In the Munich Cohort, patients admitted to the German Heart Center, Clinic at the Technical University Munich comprised 52 patients among them, 14 SARS-CoV2 qRT-PCR positive patients (5 outpatients/ER, three on normal dependency wards and six admitted to intensive care) and 38 qRT-PCR negative patients (11 outpatients/ER, 22 on standard wards and five admitted to intensive care, “Munich” cohort). COVID-19 patients in ICU were transferred from other secondary or tertiary care hospitals to the German Heart Center with need for intensive and ventilation support care. Four patients received additional Extracorporeal Membrane Oxygenation therapy (ECMO). All other patients were admitted to the hospital with suspicion of SARS-CoV-2 infection and entered the corona check-up with RT-PCR analysis-with at least two genes (E-gene and ORF-1a gene) being tested—clinical and eventually imaging exams, e.g., computed tomography, as well as routine lab diagnostics: 16 of them in the outpatients or emergency unit and 25 at the standard wards. The study was reviewed and approved by the IRB of the Technical University Munich (No 511/20S).

Blood was taken from patients as part of routine diagnostic setting for an extended corona lab profile including standard routine parameters and new markers for organ damage and inflammation such as cytokeratins, cytokines and diverse inflammatory protein markers. EDTA-samples were collected in 2.7 ml K3E EDTA-tubes (Sarstedt, Nürmbrecht, Germany) and routine blood count was done within 1 h after venipuncture. Subsequently, samples were centrifuged at 2,000 g for 10 min at room temperature. Plasma was aliquoted into 0.5 ml cryotubes and stored at −80°C in temperature-controlled freezers until use. Further markers CRP on the Cobas C501 and IL-6 on the Cobas E411 analyzer (Roche Diagnostics, Mannheim, Germany) were done as parts of the corona lab profile, too.

### Assays

Levels of circulating H3.1 nucleosomes and H3R8cit nucleosomes were measured using Nu.Q™ ELISA assays (Belgian Volition SRL, Isnes, Belgium) according to the manufacturer’s instruction. Briefly, plasma samples (20 µl in duplicate) were incubated for 2 h 30°C at room temperature in a 96-well microtiter plate coated with a monoclonal antibody raised again either a Histone H3.1 or a Histone H3R8cit epitope. After washing steps, the level of nucleosomes was quantified by adding 100 µl of a HRP-labelled anti-nucleosome detection antibody directed to a nucleosome conformational epitope (incubation 90 min at room temperature). The wells were washed and a peroxidase substrate: 3,3’,5,5’-Tetramethylbenzidine (TMB) was added. After 20°min, the colorimetric reaction was stopped by adding 100 µ1 of Stop solution. The optical densities of the well were read at 450 nm using a microplate reader (Multiskan™, Thermo Fisher Scientific, Inc.). In the Munich Biomarker Research Center of the German Heart Center ELISAs were performed using the automated ELISA processing system DS2 (Dynex Technologies, Denkendorf, Germany). Results were evaluated using Microsoft Excel and Analyze IT software (Analyse-it Software, Leeds, United Kingdom) with weighted calibration curves and significance for biomarker correlations were performed according to Best and Roberts (Best and Roberts, 1975).

## Data Availability

The raw data supporting the conclusions of this article will be made available by the authors, without undue reservation.
