# Supplementary material for: Circulating Nucleosomes as Potential Markers to Monitor COVID-19 Disease Progression
Source: Front Mol Biosci. 2021 Mar 18;8:600881. doi: 10.3389/fmolb.2021.600881 (PMC8012533; doi:10.3389/fmolb.2021.600881)
Supplement: Supplementary file 1 [file datasheet1.pdf]

Circulating Nucleosomes as Potential Markers to Monitor COVID-19 Disease Progression

Etienne Cavalier[1*], Julien Guiot[2*], Katharina Lechner[3,4], Alexander Dutsch[3,4,] Mark Eccleston[5], Marielle Herzog[5], Thomas Bygott[5], Adrian Schomburg[6,7,8], Theresa Kelly[9*], Stefan Holdenrieder[10*]

Supplementary Figure 1

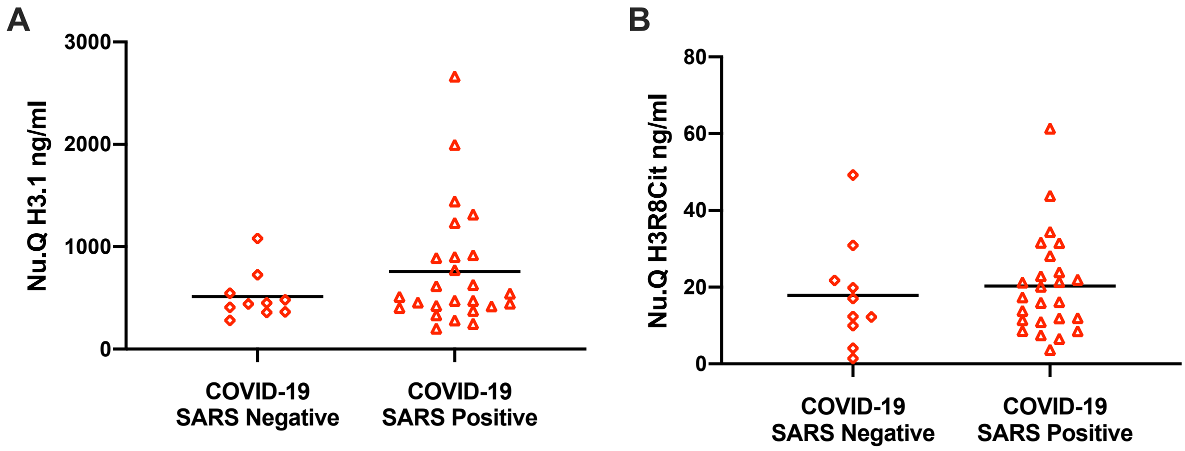

Supplementary Figure 1: Detection of circulating nucleosomes associated with H3.1 or H3R8Cit in plasma of COVID-19 patients with SARS or COVID-19 patients without SARS. Plasma from COVID-19 patients with SARS (n=25) and COVID-19 patients without SARS (n=10) were assayed with Nu.Q H3.1 (A) or Nu.Q H3R8Cit (B). COVID-19 patients were compared using a Wilcoxon-Mann-Whitney test and not significantly different from each other.
